# Elevated monocyte-to-HDL cholesterol ratio predicts post-stroke depression

**DOI:** 10.3389/fpsyt.2022.902022

**Published:** 2022-07-22

**Authors:** Yaqiang Li, Mei Zhang, Min Xue, Dalei Liu, Jinglong Sun

**Affiliations:** ^1^Department of Neurology, First Affiliated Hospital of Anhui University of Science and Technology, First People’s Hospital of Huainan, Huainan, China; ^2^Department of Neurology, People’s Hospital of Lixin County, Bozhou, China

**Keywords:** inflammation, Monocyte-to-HDL Cholesterol Ratio, depression, stroke, monocyte

## Abstract

**Objectives:**

Inflammation plays an important role in the development of depression after stroke. Monocyte-to-HDL Cholesterol Ratio (MHR) recently emerged as a novel comprehensive inflammatory indicator in recent years. This study aimed to investigate whether there is a relationship between MHR levels and post-stroke depression (PSD).

**Methods:**

From February 2019 to September 2021, patients with acute ischemic stroke (AIS) were recruited within 7 days post-stroke from the two centers and blood samples were collected after admission. The 17-item Hamilton Depression Scale (HAMD-17) was used to measure depressive symptoms at 3 months after stroke. Patients were given the DSM-V criteria for diagnosis of PSD.

**Results:**

Of the 411 enrolled patients, 92 (22.38%) patients were diagnosed with PSD at 3-months follow-up. The results also showed significantly higher level of MHR in patients with depression [0.81 (IQR 0.67–0.87) vs. 0.61 (IQR 0.44–0.82), *P* < 0.001] at admission than patients without depression. Multivariate logistic regression revealed that MHR (OR 6.568, 95% CI: 2.123–14.565, *P* = 0.015) was an independent risk factor for the depression at 3 months after stroke. After adjustment for potential confounding factors, the odds ratio of PSD was 5.018 (95% CI: 1.694–14.867, *P* = 0.004) for the highest tertile of MHR compared with the lowest tertile. Based on the ROC curve, the optimal cut-off value of MHR as an indicator for prediction of PSD was projected to be 0.55, which yielded a sensitivity of 87% and a specificity of 68.3%, with the area under the curve at 0.660 (95% CI: 0.683–0.781; *P* = 0.003).

**Conclusion:**

Elevated level of MHR was associated with PSD at 3 months, suggesting that MHR might be a useful Inflammatory markers to predict depression after stroke.

## Introduction

Post-stroke depression (PSD) is one of the most frequent psychosomatic disorders after stroke (1). It occurs mostly a year after stroke onset and is particularly common within 3 months, with clinical manifestations such as depression or anxiety, lack of emotional control, reduced initiative, lack of interest, insomnia, and cognitive dysfunction (2). The prevalence of PSD ranges from 25 to 79%, with an increasing trend within 10 years after stroke onset and a 5-year cumulative prevalence of 39 to 52% (3, 4). Accumulating studies revealed that a strong association between PSD with poor functional outcome, increased mortality and lower quality of life (5, 6). Early risk assessment of PSD is vital for identifying patients with the greatest likelihood of developing PSD and providing them with optimal treatment. The current knowledge of the molecular biological mechanisms that could lead to depression, involving the roles of inflammatory response, oxidative stress, and nitrosative stress pathways; neurotransmitter systems; neurotrophin and regulation of neurogenesis; and modulation of the hypothalamic-pituitary-adrenal axis, has been comprehensively reviewed (7, 8). Previous studies showed pro-inflammatory cytokines were involved both in the inflammatory response to acute ischemic stroke (AIS) and depression (9). A recent review also indicated that inflammation involves a significant role in the process of stroke rehabilitation and development of PSD (10). Extensive investigations showed that high levels of inflammatory and pro-inflammatory markers were involved in the inflammatory response to PSD, including neutrophil-to-lymphocyte (NLR), platelet-to-lymphocyte (PLR), tumor necrosis factor-α (TNF-α), interleukin (IL)-6, IL-18, high-sensitivity C-reactive protein (hs-CRP) and macrophage inflammatory protein 1α (11–16). Therefore, it is considered that the above systemic inflammatory processes may act role in the pathophysiology of PSD.

Monocytes/macrophages are the cell type which exerts a vital role in the release of pro-inflammatory cytokines and are involved in all phases of the inflammatory process (17). They can contribute to inflammatory responses and reduce plaque stability through the release of pro-inflammatory cytokines, leading to complications such as plaque rupture and hemorrhage and thrombosis (18, 19). Apart from that, monocytes/macrophages have also been found to be associated with stroke-induced inflammation and injury after ischemic stroke events (20). On the contrary, HDL cholesterol is known to reduce the risk of atherosclerotic events by reversing cholesterol transport and preventing endothelial dysfunction, producing anti-apoptotic, antioxidant, anti-inflammatory and anti-thrombotic effects (21). Furthermore, HDL plays an anti-atherogenic role by controlling the activation of monocytes and the proliferation of monocyte precursor cells, as well as by inhibiting the migration of macrophages and the oxidation of LDL and protecting endothelial cells from inflammation and oxidative stress (22).

A recent review also indicated that MHR is a prognostic marker in cardiovascular diseases (23, 24). Min et al. found that higher MHR levels were related to presence and severity of obstructive sleep apnea in hypertensive patients (25). You et al. reported that higher MHR was associated with increased risk of disability or death at 3 months after intracerebral hemorrhage (23). Moreover, Bolayir et al. demonstrated that a high MHR value was an independent predictor of 30-day mortality in patients with AIS (26). Up until now, no study has explored the relationship between MHR and the depression after stroke. Considering that PSD is a well-known state of systematic inflammation, we assumed that the MHR could serve as a predictor of patients with PSD. Therefore, the aim of the study was to investigate the association between MHR and PSD and to further explore the predictive value of MHR in depression at 3 months after stroke.

## Materials and methods

### Subjects

**FIGURE 1:**
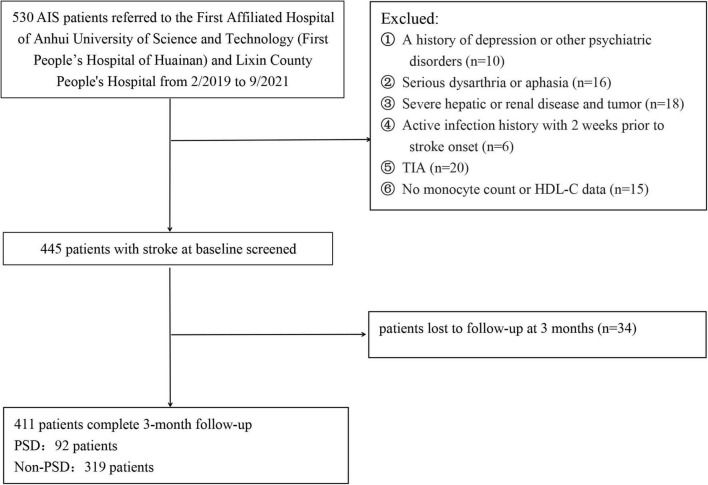
Flowchart of participant selection.

Patients with acute ischemic stroke (AIS) were consecutively enrolled from the First Affiliated Hospital of Anhui University of Science and Technology (First People’s Hospital of Huainan) and Lixin County People’s Hospital from February 2019 to September 2021. Patients were not included if they: (1) had a history of depression or other psychiatric disorders; (2) had serious dysarthria or aphasia; (3) had severe hepatic or renal disease and tumor; (4) had active infection history with 2 weeks prior to stroke onset; (5) were diagnosed with transient ischemic attacks (TIA); (6) had no serum monocyte count or HDL-C data; (7) were loss of follow-up at 3 months; and (8) were diagnosed with obstructive sleep apnea hypopnea syndrome (OSAHS). Ultimately, 414 patients with AIS were included in this study (Figure 1). This study was approved by the ethic committee of the two participating centers, and all participants or their immediate family members signed an informed consent form before participation, according to the Helsinki Declaration of 1975.

### Data collection

We collected baseline information, including patient demographics (gender, age, marital status, level of education), vascular risk factor (hypertension, diabetes mellitus, coronary heart disease, atrial fibrillation, current smoking, alcohol consumption, prior stroke), and imaging data (computed tomography or magnetic resonance imaging). AIS severity was assessed by certified neurologist using the National Institutes of Health Stroke Scale (NIHSS) on the first day after admission (27). The Barthel index (BI), modified Rankin Scale (mRS), and the Mini-Mental State Exam (MMSE) were used to evaluate functional outcome and cognitive functions, respectively, at 3 months follow-up (28–30).

### Blood collection and laboratory test

We recruited participators with AIS within 7 days of stroke onset. Blood samples were collected from the patients in the morning after 8 h of fasting. The WBC and monocyte were determined by the Sysmex XE-2100 Hematology Automated Analyzer (Sysmex, Kobe, Japan) at our hospital’s laboratory by investigators blinded to the clinical outcomes and neuroimaging findings. Glucose, total cholesterol (TC), triglycerides (TG), high density lipoprotein cholesterol (HDL-C), low density lipoprotein-cholesterol (LDL-C), homocysteine (Hcy), apolipoprotein A (ApoA), apolipoprotein B (ApoB) were analyzed with AU5811 automatic biochemical analyzer (Beckman Coulter, United States). MHR was calculated as the monocyte count (10^9^/L)/HDL cholesterol (mmol/L). All methods and procedures used in this study followed the relevant instructions strictly.

### Psychological measurement

All of the patients were interviewed or telephone interviews for depressive symptoms by the 17-item Hamilton Depression Scale (HAMD-17) at the 3 month follow-up (31). Patients with a HAMD-17 score of > 7 were diagnosed with PSD. The diagnosis of PSD was in accordance with the Diagnostic and Statistical Manual of Mental Disorders, 5th edition (DSM-V) criteria for depression.

Neuropsychological scale evaluation was performed by two trained neurological physicians who were blinded to the patients’ laboratory results.

### Statistical analysis

Statistical analyses were performed with SPSS 22.0 statistical software (SPSS Inc., Chicago, IL, United States). Data for continuous variables were shown as mean ± standard deviation (SD) or median [interquartile range (IQR)], depending on the normal or non-normal distribution of data tested by Kolmogorov-Smirnov test. Comparison of categorical variables were analyzed by chi-square test. When the continuous variables were tested for abnormal distribution, the Kruskal-Wallis test and Mann-Whitney *U* test were used to assess differences in three-group and two-group comparisons, respectively. The continuous variables of normal distribution were analyzed with Student’s *t*-test or one-way analysis of variance (ANOVA). Multivariate logistic regression analysis was performed to determine the independent risk factors for the depression at 3 months after stroke. Pearson or Spearman rank correlation analyses were used to evaluate the linear correlations between HAMD scores and other factors (NIHSS score and mRS score). Patients were divided into tertiles based on admission MHR (Q1 ≤ 0.51, Q2 0.52–0.81, Q3 ≥ 0.82). Multivariable regression analyses were performed using 3 models to recognize the predictive factors of PSD, among which, model 1 was adjusted for age and sex; model 2 was adjusted for model 1 and vascular risk factors model 3 was further adjusted for variables with *P* < 0.05 upon univariate analysis (basal ganglia lesions, baseline NIHSS score, mRS score, HDL-C and monocyte). Besides, the association was indicated as odds ratio (OR) with 95% confidence interval (CI). In addition, a receiver operating characteristic (ROC) curve analysis was used to identify the cutoff point on the MHR levels on admission with the greatest sensitivity and specificity to predict PSD at the 3-months follow-up. The area under the curve (AUC) of MHR levels was calculated as a measurement of the accuracy of the test. Statistical significance was considered as *P* < 0.05.

## Results

### Baseline characteristics of patients in post-stroke depression group and non-post-stroke depression group

Patients with AIS at the two participating centers between February 2019 and September 2021 were admitted in the study. Initially, 530 participants were enrolled, but 119 participants were excluded from the study because 34 participants were lack of follow-up at 3 months, 15 participants were lack of monocyte count or HDL data, and 70 participants met the exclusion criteria, such as TIA, infection, a history of depression or other psychiatric disorders, serious dysarthria or aphasia, hepatic or renal disease and tumor, etc. Finally, 411 patients were included in the study (232 males, aged 66.27 ± 8.43 years), including 92 (22.38%) patients with PSD and 319 (77.62%) patients without PSD.

Table 1 shows the patients’ demographic characteristics, vascular risk factors, lesion location, neuropsychological function and laboratory data according to the presence or absence of PSD. In this study, 92 (22.38%) patients were diagnosed with PSD at 3-months follow-up. Compared with subjects without PSD, those with PSD had higher proportions of female (*P* = 0.033), higher proportions of basal ganglia lesions (*P* = 0.015), higher baseline NIHISS score (*P* < 0.001), higher mRS score (*P* < 0.001), lower BI score (*P* < 0.001), lower HDL-C (*P* = 0.043), higher levels of monocyte (*P* = 0.009) and MHR (*P* < 0.001).

**TABLE 1:** Clinical and demographic characteristics of patients with PSD and non-PSD.

Variables	Total (*n* = 411)	Depression after stroke		*P*- value
		Depression (*n* = 92)	Non-depression (*n* = 319)	
**Demographic characteristics**				
Gender, female, n (%)	179 (43.55)	50 (54.34)	129 (41.75)	0.033
Age, years, mean ± SD	66.27 ± 8.43	65.64 ± 9.2	66.45 ± 8.3	0.421
Education years, median (IQR)	5 (3–8)	5 (3–8)	4 (2–7)	0.870
Married, n (%)	387 (94.16)	86 (62.77)	301 (94.36)	0.751
**Vascular risk factors (%)**				
Hypertension	268 (65.21)	63 (68.48)	205 (64.26)	0.455
Diabetes mellitus	132 (32.12)	34 (36.96)	98 (30.72)	0.259
Coronary heart disease	52 (12.65)	13 (14.13)	39 (12.23)	0.628
Atrial fibrillation	19 (4.62)	5 (5.43)	14 (4.39)	0.674
current smoking	111 (27.01)	24 (26.09)	87 (27.27)	0.821
Alcohol consumption	132 (32.12)	30 (32.61)	102 (31.97)	0.909
Prior stroke	77 (18.73)	17 (17.48)	60 (18.81)	0.943
**Laboratory findings (IQR)**				
WBC, × 10^9^/L, median (IQR)	6.35 (5.28–7.53)	6.4 (5.59–7.73)	6.33 (5.24–7.50)	0.225
Monocyte, × 10^9^/L, median (IQR)	0.64 (0.46–0.81)	0.71 (0.54–0.86)	0.61 (0.44–0.79)	0.009
Glucose (mmol/L)	5.3 (4.7–6.8)	5.4 (4.80–7.48)	5.2 (4.7–6.7)	0.173
TG, mmol/L	1.36 (0.95–1.93)	1.43 (0.97–1.91)	1.34 (0.94–1.99)	0.780
TC, mmol/L	4.51 (3.78–5.35)	4.51 (3.81–5.32)	4.48 (3.67–5.47)	0.797
HDL-C, mmol/L	1.01 (0.84–1.22)	0.99 (0.82–1.15)	1.04 (0.86–1.27)	0.043
LDL-C, mmol/L	2.52 (1.96–3.17)	2.48 (1.97–3.33)	2.51 (1.93–3.16)	0.521
ApoA, g/L	1.26 (1.10–1.43)	1.28 (1.12–1.43)	1.26 (1.09–1.44)	0.315
ApoB, g/L	0.86 (0.69–1.02)	0.85 (0.67–1.11)	0.86 (0.69–1.0)	0.626
Hcy, μmol/L	12.6 (10.24–16.31)	13.09 (10.95–17.15)	12.32 (10.02–16.18)	0.074
MHR	0.65 (0.47–0.87)	0.81 (0.67–0.87)	0.61 (0.44–0.82)	< 0.001
**Lesion location, n (%)**				
Frontal lobe	78 (18.98)	20 (21.74)	58 (18.18)	0.443
Parietal lobe	78 (18.98)	15 (16.30)	63 (19.75)	0.458
Temporal lobe	87 (21.17)	25 (27.17)	62 (19.44)	0.109
Occipital lobe	60 (14.60)	11 (11.96)	49 (15.36)	0.415
Basal ganglia	111 (27.01)	34 (36.90)	77 (24.14)	0.015
Brainstem	76 (18.49)	20 (21.74)	56 (17.55)	0.362
Cerebellum	25 (21.74)	9 (9.78)	16 (21.74)	0.092
**Neuropsychological function**				
NIHSS score, median (IQR)	6 (4–9)	8 (6–10)	6 (4–8)	< 0.001
MMSE score, median (IQR)	22 (17–26)	21 (17–25)	22 (17–26)	0.380
BI score, median (IQR)	75 (50–90)	60 (40–75)	80 (60–95)	< 0.001
mRS score, median (IQR)	3 (2–4)	3 (3–4)	2 (2–3)	< 0.001
HAMD score, median (IQR)	5 (2–7)	12 (9–18)	3 (2–5)	< 0.001

WBC, White blood cell; TG, total triglyceride; TC, total cholesterol; LDL-C, low density lipoprotein cholesterol; HDL-C, high density lipoprotein cholesterol; ApoA, apolipoprotein A; ApoB, apolipoprotein B; Hcy, homocysteine; MHR, Monocyte-to-HDL Cholesterol Ratio; mRS, modified Rankin Scale; NIHSS, National Institute of Health Stroke Scale; MMSE, Mini-Mental State Examination; IQR, inter-quartile range; BI, Barthel index; HAMD, Hamilton depression scale.

### Baseline characteristics of all patients in monocyte-to-HDL cholesterol ratio tertiles

All patients have been divided into three subgroups in accordance to tertiles of the levels of MHR, which ensured the most categories with sufficient variety of patients per subgroups from the range of 0.23 to 0.97 (Q1, 137 patients; Q2, 137 patients; Q3, 127 patients). The cut-off values for this stratification of the MHR into tertiles were: (Q1) 0.23–0.51, (Q2) 0.52–0.81, (Q3) 0.82–0.97. Table 2 illustrates ascending tertiles of MHR was associated with lower HDL-C (*P* = 0.002), higher monocyte count (*P* < 0.001), basal ganglia (*P* = 0.001), also with higher NIHSS score (*P* = 0.002) and higher mRS score (*P* = 0.036). There were significant differences between the PSD and non-PSD groups in MHR tertiles of patients (*χ^2^* = 23.47, *P* < 0.001). Indeed, the percentage of patients in the lowest tertiles (0.23–0.51) was significantly lower in the PSD group, while the percentage of patients in the highest tertiles (0.82–0.97) was notably higher in the PSD group. Besides, the numbers of patients with depression after stroke were 12 (13.04%), 35 (38.04%), and 45 (48.91%) in Tertile 1, Tertile 2, and Tertile 3, respectively (Table 3).

**TABLE 2:** Baseline characteristics of patients with AIS according to MHR tertiles.

Variables	MHR			*P*- value
	Q1 (≤0.51, *n* = 137)	Q2 (0.52–0.81, *n* = 137)	Q3 (≥0.82, *n* = 137)	
**Demographic characteristics**				
Gender, female, n (%)	69 (50.36)	58 (42.34)	52 (37.96)	0.110
Age, years, mean ± SD	67.3 ± 8.18	65.95 ± 8.41	65.55 ± 8.67	0.105
Education years, median (IQR)	5 (3–7)	6 (3–8)	5 (3–8)	0.196
Married, n (%)	132 (93.48)	128 (93.48)	127 (93.48)	0.395
**Vascular risk factors (%)**				
Hypertension	88 (64.23)	96 (70.07)	83 (60.58)	0.252
Diabetes mellitus	53 (38.69)	41 (29.93)	38 (27.74)	0.121
Coronary heart disease	15 (10.95)	20 (14.60)	17 (12.41)	0.658
Atrial fibrillation	6 (4.38)	6 (4.38)	7 (5.11)	0.946
current smoking	38 (27.74)	26 (18.98)	30 (21.90)	0.213
Alcohol consumption	44 (32.12)	52 (37.96)	36 (26.28)	0.117
Prior stroke	29 (21.17)	22 (16.06)	26 (18.98)	0.554
**Laboratory findings (IQR)**				
WBC, × 10^9^/L, median (IQR)	6.24 (5.25–7.37)	6.15 (5.22–7.49)	6.68 (5.55–7.77)	0.180
Monocyte, × 10^9^/L, median (IQR)	0.45 (0.35–0.55)	0.64 (0.53–0.80)	0.81 (0.69–0.96)	< 0.001
Glucose (mmol/L)	5.40 (4.70–7.90)	5.41 (4.8–6.9)	5.2 (4.7–6.1)	0.237
TG, mmol/L	1.32 (0.91–1.83)	1.35 (0.92–1.93)	1.41 (1.02–2.08)	0.325
TC, mmol/L	4.54 (3.80–5.34)	4.51 (3.62–5.54)	4.50 (3.92–5.17)	0.872
HDL-C, mmol/L	10.6 (0.92–1.38)	1.02 (0.82–1.26)	0.98 (0.81–1.17)	0.002
LDL-C, mmol/L	2.49 (1.96–3.18)	2.52 (1.81–3.21)	2.51 (1.99–3.07)	0.955
ApoA, g/L	1.26 (1.12–1.43)	1.25 (1.07–1.48)	1.26 (1.11–1.42)	0.911
ApoB, g/L	0.84 (0.7–1.06)	0.89 (0.67–0.99)	0.87 (0.87–0.89)	0.873
Hcy, μmol/L	12.61 (9.94–16.30)	11.82 (10.02–15.74)	13.01 (10.87–16.55)	0.074
MHR	0.43 (0.35–0.47)	0.65 (0.61–0.67)	0.87 (0.87–0.89)	< 0.001
**Lesion location, n (%)**				
Frontal lobe	26 (18.98)	29 (21.17)	23 (16.79)	0.652
Parietal lobe	30 (21.89)	27 (19.70)	21 (15.33)	0.369
Temporal lobe	21 (15.33)	37 (27.01)	29 (21.17)	0.061
Occipital lobe	16 (11.68)	23 (16.79)	21 (15.33)	0.467
Basal ganglia	50 (36.49)	22 (16.06)	39 (28.47)	0.001
Brainstem	24 (17.52)	23 (16.79)	29 (21.17)	0.606
Cerebellum	5 (3.65)	8 (5.84)	12 (8.76)	0.207
**Neuropsychological function**				
NIHSS score, median (IQR)	6 (4–8)	7 (5–9)	7 (4–10)	0.022
MMSE score, median (IQR)	22 (17–26)	23 (17–26)	21 (17–25)	0.718
BI score, median (IQR)	80 (50–95)	70 (50–90)	70 (45–90)	0.071
mRS score, median (IQR)	2 (2–3)	3 (2–4)	3 (2–4)	0.036
HAMD score, median (IQR)	5 (2–6)	5 (3–8)	5 (2–9)	0.111

WBC, White blood cell; TG, total triglyceride; TC, total cholesterol; LDL-C, low density lipoprotein cholesterol; HDL-C, high density lipoprotein cholesterol; ApoA, apolipoprotein A; ApoB, apolipoprotein B; Hcy, homocysteine; MHR, Monocyte-to-HDL Cholesterol Ratio; mRS, modified Rankin Scale; NIHSS, National Institute of Health Stroke Scale; MMSE, Mini-Mental State Examination; IQR, inter-quartile range; BI, Barthel index; HAMD, Hamilton depression scale.

**TABLE 3:** MHR tertiles of patients.

Variables	Depression (*n* = 92)	Non-depression (*n* = 319)	*χ^2^*	*P*- value
**MHR**				< 0.001
Tertile 1 (0.35–0.47)	12 (13.04%)	125 (32.98%)	21.96	< 0.001
Tertile 2 (0.61–0.67)	35 (38.04%)	102 (31.97%)	1.183	0.227
Tertile 3 (0.87–0.89)	45 (48.91%)	92 (28.84%)	12.95	< 0.001

MHR, Monocyte-to-HDL Cholesterol Ratio.

### Association between the level of monocyte-to-HDL cholesterol ratio and post-stroke depression

Gender, monocyte, basal ganglia infarction, HDL-C, MHR, NIHSS score, mRS score, and BI score were included in the multivariate analysis as independent variables. Multivariate logistic regression revealed that mRS score (OR 2.480, 95% CI: 1.246–4.933, *P* = 0.048), NIHSS score (OR 1.345, 95% CI: 1.182–1.934, *P* = 0.035), MHR (OR 6.568, 95% CI: 2.123–14.565, *P* = 0.015) were independent risk factors for the depression at 3 months after stroke (Table 4). Correlation analyses revealed that NIHISS scores were positively correlated with the HAMD scores 3 months after admission in all patients (*r* = 0.288, *P* < 0.001), and mRS scores were also positively correlated with HAMD scores 3 months after admission in all patients (*r* = 0.197, *P* < 0.001).

**TABLE 4:** Multivariate logistic regression analysis for depression in stroke patients.

Variables	OR	95% CI	*P*
Gender (female)	1.268	0.365–4.786	0.238
Monocyte	4.895	0.830–14.875	0.079
Basal ganglia	0.761	0.329–1.957	0.076
HDL-C	0.818	0.215–3.119	0.087
MHR	6.568	2.123–14.565	0.015
mRS score	2.480	1.246–4.933	0.048
BI score	0.326	0.179–0.924	0.067
NIHISS score	1.345	1.182–1.934	0.035

MHR, Monocyte-to-HDL Cholesterol Ratio; CI, confidence interval; OR, odds radio; PSD, post-stroke depression; mRS, modified Rankin Scale; NIHSS, National Institute of Health Stroke Scale; BI, Barthel index.

**FIGURE 2:**
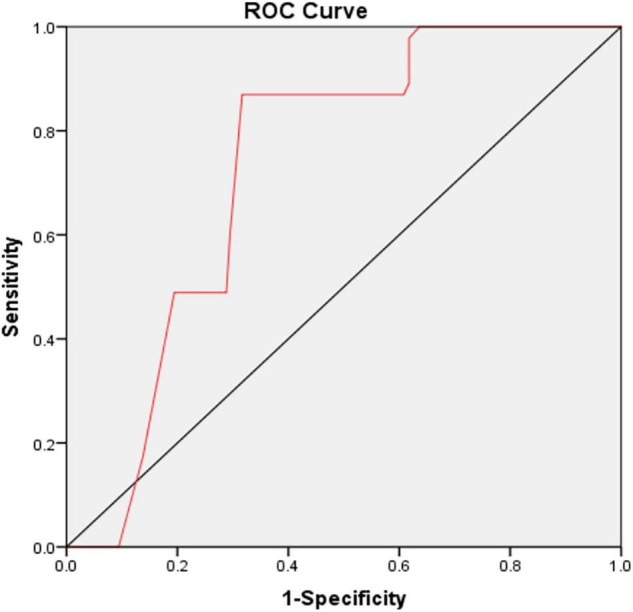
The ROC curve analysis for MHR in predicting PSD. The cut-off value of MHR was 0.55, with a sensitivity of 87% and a specificity of 68.3%, AUC: 0.660 (95% CI: 0.683–0.781; *P* = 0.003). ROC, receiver operating characteristic; MHR, monocyte to high-density lipoprotein cholesterol ratio; PSD, post-stroke depression; AUC, area under the curve; CI, confidence interval.

In Table 5, with all patients taken as a whole, PSD occurrence taken as a dependent variable and lowest tertile taken as the reference was used for MHR in the unadjusted and multivariate-adjusted logistic regression models. In the unadjusted logistic regression model, PSD was significantly higher among study participants with admission MHR in the highest quartile compared with those in the lowest tertile (non-adjusted: OR 5.095, 95% CI: 2.552–10.172, *P* < 0.001). And after adjusting for confounders including age, sex, education years, married, vascular risk factors (hypertension, diabetes mellitus, coronary heart disease, atrial fibrillation, current smoking, alcohol consumption, prior stroke), basal ganglia lesions, baseline NIHSS score, mRS score and laboratory data (HDL-C, monocyte), the highest tertile of MHR was remained significant independently associated with the prevalence of PSD (model 1: OR = 5.853, 95% CI = 2.880–11.893, *P* < 0.001; model 2: OR = 6.742, 95% CI = 3.252–13.976, *P* < 0.001; model 3: OR = 5.018, 95% CI = 1.694–14.867, *P* = 0.004). Based on the ROC curve, the optimal cut-off value of MHR as an indicator for prediction of PSD was projected to be 0.55, which yielded a sensitivity of 87% and a specificity of 68.3%, with the area under the curve at 0.660 (95% CI: 0.683–0.781; *P* = 0.003, Figure 2).

**TABLE 5:** Multivariate logistic regression analysis model of MHR and PSD.

	Tertile	OR^a^	95% CI	*P* value
Unadjusted	Middle	1.425	0.844–2.407	0.185
	Highest	5.095	2.552–10.172	*P* < 0.001
Model 1^b^	Middle	1.491	0.874–2.544	0.143
	Highest	5.853	2.880–11.893	*P* < 0.001
Model 2^c^	Middle	1.684	0.970–2.924	0.064
	Highest	6.742	3.252–13.976	*P* < 0.001
Model 3^d^	Middle	1.669	0.851–3.272	0.136
	Highest	5.018	1.694–14.867	0.004

MHR, Monocyte-to-HDL Cholesterol Ratio; CI, confidence interval; OR, odds radio; CI, confidence level; PSD, post-stroke depression.

^a^Reference OR (1.000) is the lowest tertile of MHR for PSD.

^b^Model 1: adjusted for age, sex, education years, married.

^c^Model 2: adjusted for covariates from Model 1 and further adjusted for Vascular risk factors (Hypertension, diabetes mellitus, coronary heart disease, atrial fibrillation, current smoking, alcohol consumption, prior stroke).

^d^Model 3: adjusted for covariates from Model 2 and further adjusted for variables with P < 0.05 in univariate analysis (basal ganglia lesions, baseline NIHSS score, mRS score).

## Discussion

Our results observed high levels of MHR at admission were significantly correlated with depression at 3 months after stroke and documented a 5.018-fold higher risk of PSD among individuals with the highest tertile of MHR compared with those with the lowest tertile of MHR after adjustment for major confounders. To our best knowledge, this is the first article in the literature that evaluates the prospective association of MHR levels with the PSD. Therefore, our results first revealed the MHR value is a potentially important biological marker of risk for the PSD.

Indeed, a total of 22.38% of AIS patients were diagnosed as depression at 3 months in this study, which was consistent with the incidence reported in previous studies (32). Our results also revealed that stroke severity and functional impairment were risk factors for the development of PSD, which was broadly consistent with the findings of earlier studies (3). The significant association between the development of PSD and lesion location was found, although previous studies have provided inconclusive results on the aspect. We observed that the location of the basal ganglia lesions may promote the occurrence of depression. The basal nuclei or the located in the frontal region are the core brain areas of the emotional network, and any damage in these brain areas will contribute to depressive symptoms (33). Consistently, Chatterjee et al. had demonstrated a close relationship between basal ganglia lesions and affective symptoms, including depression (34).

An increasing amount of evidence has shown that immune inflammatory responses and oxidative stress play an important role in stroke (35, 36). Monocytes, a specific subtype of leukocytes, participate in the inflammatory damage response by binding to adhesion molecules on activated endothelial cells and infiltrating into the central nervous system in the acute phase after ischemic stroke, and can appear as a tissue cell infiltration within 4 h after an acute ischemic attack, reaching a peak after 7 days (35, 37). Under the stimulation of oxidized LDL, monocytes adhere to endothelial cells and can differentiate into macrophages, which infiltrate the arterial wall and interact with endothelial cells by producing various types of pro-inflammatory and pro-oxidant mediators, leading to an inflammatory cascade response and foam cell formation through phagocytosis of oxidized LDL and other lipids, which then evolve into atherosclerosis or atherosclerotic plaques (38, 39). Similarly, it also has been shown that monocytes are associated with atherosclerotic plaques and predict plaque progression and plaque volume (40). The overexpression of monocyte chemoattractant protein-1 (MCP-1) exacerbates brain injury by recruiting inflammatory cells including pro-inflammatory cytokines such as IL-6, IL-1β, TNF-α, and granulocyte colony-stimulating factor (41). Liberale et al. had reported that monocyte can predict worse post-stroke outcome during a 90-day follow-up (42). Besides, it is well demonstrated that monocytes have three subpopulations and circulate in a dynamic equilibrium, including classical monocytes, intermediate monocytes and non-classical monocytes (43). Classical monocytes lead to ischemic lesions by producing IL-1β, IL-6, and TNF-α, which are connected with the presence of depression (44–47). Alvarez-Mon et al. reported that intermediate monocytes also play a proinflammatory role in the development of major depressive disorder (MDD) by secreting IL-1β and IL-6 (47). Non-classical monocytes act a dual role in the inflammatory process in MDD, releasing IL-10 to promote recovery of MDD patients and aggravating the progression of depression by secreting IL-6 and IL-12 (48, 49). Additionally, a recent study also reported that increased immune cell counts, especially monocytes, were associated with increased risk of MDD (50). Therefore, given its involvement in stroke as well as depression, we hypothesized that monocyte subpopulations shift over time after stroke and play a vital role in the development of PSD.

It is well showed that HDL-C plays a protective role in the development of atherosclerosis and is a vascular protective factor (51). HDL-C exerts its anti-inflammatory effects by inhibiting the activation of CD11b and HDL-C inhibition may be related to the signaling pathway mediated by scavenger class B and sphingosine type 1 receptors (52). In addition to inhibiting the migration and adhesion of monocytes, HDL-C also promotes cholesterol efflux from cells and inhibits the oxidation of lipoproteins, which inhibits thrombosis and effectively blocks oxidative stress and inflammatory responses (53, 54). HDL can remove cholesterol from the vascular wall, control plaque progression, stabilize plaque, and reduce the occurrence of acute cerebral infarction. Besides, serum HDL-C had been shown to be significantly related to inflammatory markers, including albumin and serum zinc (55). In another study, lower levels of HDL-C were reported to be partly a result of low-grade inflammation, which was mediated by IL-6 and has been found in depression (56). Shen et al. also found decreased levels of HDL-C are associated with depression at the 1-month follow-up (57). However, a Korean cross-sectional studies that included 8207 participants found that elevated HDL levels were significantly associated with increased risk for depressive symptoms in a community-based population (58). In this study, we observed levels of blood HDL were significantly lower in PSD patients than in non-PSD patients. It has been argued that those with higher levels of HDL are likely to be depressive, this issue remains contentious and need to be verified in the future.

Concerning the inflammatory properties of monocytes and the anti-inflammatory properties of HDL cholesterol, MHR, a new marker of systemic inflammation, could be considered as an easily available predictor for the occurrence of PSD. Previous study had showed that higher levels of MHR were associated with poor functional outcome in Acute Ischemic Stroke patients (59). The more severe the neurological damage to patients with acute cerebral infarction, the more intense the inflammatory response to their damaged brain tissues, the more unfavorable the recovery of the ischemic semidark zone and compressed brain tissue, and the worse the prognosis. Depressive symptoms were associated with poor functional outcome of stroke patients (60). Therefore, since decreased HDL and increased monocyte count are both found in AIS patients and depression subjects, this study clarified that AIS patients with higher MHR values were inclined to have PSD. The MHR AUC showed that MHR was a good prognostic marker for PSD, with sensitivity of 87% and specificity of 68.3%. Consequently, MHR may play a vital role to predict the occurrence of depression after stroke.

There were several limitations on interpretation of its results. First, inflammatory process has dynamics and continuity, but the MHR was not dynamically multiple times measured in our study.

The pattern of dynamic change of MHR could provide better predictable. Second, patients with severe dysarthria or aphasia were removed, which may have resulted in underestimation of the actual prevalence of PSD and contributed some bias to the results. Third, previous studies had shown that depression symptoms occur in the first month. In the present study, the depression scale was screened at 3-month follow-up. When the period of depression assessment was prolonged, it could result in an increase in the number of follow-up losses or deaths, which may affect the PSD rates. Finally, some important inflammatory factors were not routinely screened in the included patients. Therefore, more detailed and extensive prospective studies are required in the future.

## Conclusion

To sum up, a higher MHR is independently associated with depression at 3-month after stroke, which suggests that MHR on admission can serve as a significant biomarker of systemic inflammation to predict the occurrence of depression after stroke.

## Data availability statement

The raw data supporting the conclusions of this article will be made available by the authors, without undue reservation.

## Ethics statement

The studies involving human participants were reviewed and approved by the Institutional Review Board of the First People’s Hospital of Huainan (IRB number 2019-LS-031) and Lixin County People’s Hospital (IRB number LXXRMYY-2019KY01). The patients/participants provided their written informed consent to participate in this study.

## Author contributions

YL and MZ designed the research study and performed the research. YL, DL, and JS analyzed the data. YL wrote the manuscript. All authors contributed to editorial changes in the manuscript, read, and approved the final manuscript.

## Conflict of interest

The authors declare that the research was conducted in the absence of any commercial or financial relationships that could be construed as a potential conflict of interest.

## Publisher’s note

All claims expressed in this article are solely those of the authors and do not necessarily represent those of their affiliated organizations, or those of the publisher, the editors and the reviewers. Any product that may be evaluated in this article, or claim that may be made by its manufacturer, is not guaranteed or endorsed by the publisher.
